# Accurately predicting hit songs using neurophysiology and machine learning

**DOI:** 10.3389/frai.2023.1154663

**Published:** 2023-06-20

**Authors:** Sean H. Merritt, Kevin Gaffuri, Paul J. Zak

**Affiliations:** ^1^Center for Neuroeconomics Studies, Claremont Graduate University, Claremont, CA, United States; ^2^Immersion Neuroscience, Henderson, NV, United States

**Keywords:** prediction, immersion, music, neurophysiology, classification

## Abstract

Identifying hit songs is notoriously difficult. Traditionally, song elements have been measured from large databases to identify the lyrical aspects of hits. We took a different methodological approach, measuring neurophysiologic responses to a set of songs provided by a streaming music service that identified hits and flops. We compared several statistical approaches to examine the predictive accuracy of each technique. A linear statistical model using two neural measures identified hits with 69% accuracy. Then, we created a synthetic set data and applied ensemble machine learning to capture inherent non-linearities in neural data. This model classified hit songs with 97% accuracy. Applying machine learning to the neural response to 1st min of songs accurately classified hits 82% of the time showing that the brain rapidly identifies hit music. Our results demonstrate that applying machine learning to neural data can substantially increase classification accuracy for difficult to predict market outcomes.

## Introduction

Every day, 24,000 new songs are released worldwide (Pandora, 2018). That's 168,000 new songs every week. People are drowning in choices. The surfeit of choices makes it difficult for streaming services and radio stations to identify songs to add to playlists. Music distribution channels use both human listeners and artificial intelligence models to identify new music that is likely to become a hit. Unfortunately, the accuracy of predictions has generally been low (Prey, 2018). This has been called the “Hit Song Science” problem (McFee et al., 2012). The inability to predict hits means that artists are often underpaid for their work and music labels misallocate production and marketing resources when seeking to build audiences for new music (Byun, 2016). The inability to curate desirable music also causes audiences move between platforms searching for music they enjoy (Prey, 2018).

People want new music, but generally prefer songs similar to those they already know (Ward et al., 2014; Askin and Mauskapf, 2017). Music streaming services have invested in technologies to identify and introduce new music customized to subscribers' existing playlists. Spotify does this with “Discover Weekly,” a playlist of 30 new songs subscribers receive every Monday morning. Pandora classifies new music using 450 attributes in its Music Genome Project and introduces new music using a service called “Personalized Soundtracks” (Carbone, 2021). Tracking what people add to their playlists boosts the likelihood of songs showing up in related playlists thereby building support leading to a hit (Turk, 2021). Nevertheless, less than 4% of new songs will become hits (Interiano et al., 2018).

## Background

Predicting hits in entertainment is a long-standing problem (Litman, 1983). Predicting hit movies has been no better than a coin flip even after considering the director, the stars, budget, time of year of release, and whether or not the movie is a sequel (Chang and Ki, 2005; Sharda and Delen, 2006; Lash and Zhao, 2016). Various methods have been used to predict hit music, including the analysis of lyrics, blog postings, social media mentions, and brain activity (Dhanaraj and Logan, 2005; Abel et al., 2010; Berns and Moore, 2012; Singhi and Brown, 2014; Araujo et al., 2017). Yet, predictive accuracy for most studies is quite low.

The Hit Song Science problem is a subset of research known as Hit Science that seeks to predict whether entertainment content will be popular (Yang et al., 2017). This approach typically extracts components of content and applies machine learning to predict hits (Ni et al., 2011). When seeking to predict songs, this approach has typically used audio elements such as tempo, time signature, length, loudness, genre, lyric sentimentality, and instrument types (Herremans et al., 2014). Various classification techniques have been applied and yet predictive accuracy continues to be low (Raza and Nanath, 2020; Shambharkar et al., 2021).

Ex post, experts offer rationale for why hits were “inevitable” (Rodman, 2020). Yet, the apparent inevitability that some entertainment will become popular is challenged by studies that use ex-ante self-report for prediction (Morton, 1996). Direct and indirect measures of self-reported “liking” poorly predict aggregate outcomes (Hazlett and Hazlett, 1999; Wolfers and Zitzewitz, 2004; Bar-Anan et al., 2010; Cyders and Coskunpinar, 2011; John et al., 2017). When assessing music, “liking” is often anchored to familiarity resulting in poor ratings for unfamiliar songs (Ward et al., 2014). Moreover, using Likert self-report scales to predict popularity may be asking too much of study participants. Music is meant to elicit emotional responses that arise outside of conscious awareness and are often poorly reported (Thomas and Diener, 1990; Robinson and Clore, 2002). One way to avoid the inaccuracy of self-report is to directly measure neurophysiologic responses to music.

Music is an effective way to influence people's emotional states (Fitch, 2006). Music and language likely co-evolved, with the first evidence of musical instruments appearing in Paleolithic bone flutes 40,000 years ago (Conard et al., 2009). Structural features of music, including melody, tempo, key, and rhythm influence the emotions people experience (Scherer and Zentner, 2001). Lyrics, which add the human voice to music, generally increase emotional responses to music, especially for sad songs (Ali and Peynircioglu, 2006; Mori and Iwanaga, 2014). Songs are so effective at influencing emotions that they work at scale to build and sustain communities. Examples include Gregorian chants, the sacred music of Bach, the Catholic Tridentine Mass sung in Latin, Navajo priest singers, the singing of monks in the Buddhist and Daoist traditions, and Pygmy honey-gathering songs (Schippers, 2018). Songs are thought to be a way to socially regulate emotional responses, thereby influencing behavior (Hou et al., 2017). Contemporary music can be similarly powerful, influencing fads such as swing dancing, country line dancing, the Macarena, and Gangnam Style, as well as cadences used at military boot camps to teach recruits to march. Hit songs, due to their broad popularity, have an outsized influence on the emotional states of people, if only temporarily. The desire to experience an emotional state may explain why some songs become hits (Schulkind et al., 1999; Schellenberg and von Scheve, 2012).

Emotional responses emanant from multiple brain regions rather than being localized to a one or a few structures (Adolphs and Anderson, 2018). In addition to the auditory cortex, music has been shown to activate brain areas associated with processing emotions (amygdala, orbitofrontal cortex) and long-term memory retrival (hippocampus) (Koelsch et al., 2006; Levitin and Tirovolas, 2009). The multiple regions of the brain activated by music mean that peripheral rather than central measures of neural activity may better capture the response of neural circuits that process emotional stimuli (Mauss and Robinson, 2009) including responses to music (Coutinho and Cangelosi, 2011; Koelsch, 2018). This is consistent with the James-Lange theory of emotion in which neurophysiologic responses induce an emotional feeling (Derryberry and Tucker, 1992; McGaugh and Cahill, 2003; Barrett, 2006; Kreibig, 2010; Barrett and Westlin, 2021). While there is no one best way to measure neurophysiologic responses to emotional stimuli, peripheral measures appear to be more robust than central measures (Golland et al., 2014). For these reasons, the study here measured peripheral rather than central neurophysiologic responses.

## State of the art

Our point of departure from the extant literature is to examine if neural measures can be used to predict hit music in order to address the Hit Song Science problem. While neural activity has been shown to add predictive power to self-reports, neural signals alone generally have poorly predictive accuracy for population outcomes (Falk et al., 2010, 2011; Berkman and Falk, 2013; Dmochowski et al., 2014; Genevsky et al., 2017). For example, a study using functional MRI to predict music popularity showed an improvement over self-report but predictive accuracy was still well-below 50% (Berns and Moore, 2012). One reason for this may be the inherent non-linearity of neural signals used as inputs into linear predictive models. While some researchers have directly modeled the nonlinear components of neural responses (Barraza et al., 2015), this is atypical.

A recent approach seeks to predict outcomes from neural data using machine learning. Machine learning more effectively integrates non-linear effects into predictions (Guixeres et al., 2017; Wei et al., 2018). Nevertheless, machine learning analyses may be subject to overfitting the data (Lemm et al., 2011). Overfitting can be reduced by using a limited the number of neural data streams (Jabbar and Khan, 2015), an approach we take here. Our analysis compares the classification accuracy of traditional linear predictive models to machine learning models using neurophysiologic measures alone.

Rather than choose a machine learning approach a priori (Raza and Nanath, 2020), we instead used an ensemble method (González et al., 2020). This approach estimates multiple individual models that are weighted and combined in order to increase predictive accuracy. Herein we apply an ensemble learning technique called bagging, also known as bootstrap aggregating, that avoids overfitting of data by reducing variance. Bagging also effectively captures high-dimensional data (Zhang and Ma, 2012) and can be used used on weak learners with high variance and low bias (Alelyani, 2021).

## Methods

In this section we detail the procedures through which the songs were chosen and market impact measures were obtained. We also describe the design of the laboratory experiment used to collect neurophysiologic data while participants listened to songs. While a commercial neurophysiology platform was used to capture neural responses, we derived two novel measures from these data that we anticipated would add insight into why some songs become hits. This section also outlines the data analytics methodology and provides a rationale for the machine learning approach we apply to predict hit songs from neurophysiologic measures.

### Participants

Thirty-three participants (47% female) were recruited from the Claremont Colleges and surrounding community. Participants ranged in age from 18 to 57 (M = 24.25, SD = 10.47). This study was approved by the Institutional Review Board of Claremont Graduate University (#3574) and all participants gave written informed consent prior to inclusion. The data were anonymized by assigning an alphanumeric code to each participant.

### Procedure

**Figure 1:**
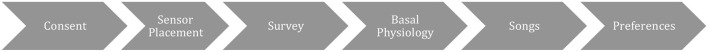
Timeline of the experiment.

After consent, participants were seated and fitted with Rhythm + PPG cardiac sensors (Scosche Industries, Oxnard, CA). Music was played through a speaker system to groups of 5–8 participants in a medium-sized lab. Participants were informed that they would listen to 24 recent songs and asked about their preferences for each one. They then completed a short survey on demographics. The study lasted ~1 h and participants were paid $15 for their time. Figure 1 shows the study timeline.

### Neurophysiology

A commercial platform (Immersion Neuroscience, Henderson, NV) was used to measure neurophysiologic responses. Neurophysiologic immersion combines signals associated with attention and emotional resonance collected at 1 Hz. The attentional response is associated with dopamine binding to the prefrontal cortex while emotional resonance is related to oxytocin release from the brainstem (Barraza and Zak, 2009; Zak and Barraza, 2018; Zak, 2020). Together these neural signals accurately predict behaviors after a stimulus, especially those that elicit emotional responses (Lin et al., 2013; Barraza et al., 2015). The Immersion Neuroscience platform ingests device-agnostic heart rate data to infer neural states from activity of the cranial nerves using the downstream effects of dopamine and oxytocin (JeŽová et al., 1985; Zak, 2012; Barraza et al., 2015). The algorithms that measure immersion from cranial nerve activity are cloud-based and the platform provides an output file used in the analysis. We chose to measure neurologic immersion for this study because singing induces oxytocin release (Keeler et al., 2015) as does listening to music (Nilsson, 2009; Ooishi et al., 2017), though the effect is inconsistent (Harvey, 2020). The Immersion omnibus measure was expected to be more predictive than oxytocin alone or peripheral neural measures such as electrodermal activity (Ribeiro et al., 2019). Whether neurologic immersion can accurately classify hit songs is a new use of this measure.

The independent variables were average immersion for each song as well as two additional variables we derived from immersion data. The first we call peak immersion, defined as


$$\overset{T}{\underset{t=0}{\int }}\left(v_{it}>M_{i}\right)d_{t}/Im_{i}$$

where ν_*it*_ is average neurophysiologic immersion across participants in song *i* at time *t* to the end of the song at time *T*, *M*_*i*_ is the median of the average time series of immersion for the duration of song *i* plus the standard deviation of song *i* across all participants who listened to that song divided by the sum of total immersion *Im*_*i*_ for song *i*. That is, peak immersion cumulates the highest immersion moments during the song relative to the song's total immersion. The second variable we created is called retreat. Neurologic retreat cumulates the lowest 20% of immersion averaged across participants for each song.

### Songs

Staff from an online streaming service choose 24 songs for this study without input from the researchers. The streaming service also provided the definition of hits or flops. This resulted in a “clean” experiment as song choice could not be cherry-picked for the study and the criterion for a hit was established in advance. Thirteen songs were deemed “hits” with over 700,000 streaming listens, while the other 11 were flops. The songs had been released for no more than 6 months and spanned genres that included rock (Girl In Red “Bad Idea”), hip-hop (Roddy Rich “The Box”), and EDM (Tons and I “Dance Monkey”). Song order was counterbalanced and song start and stop times were synchronized with physiologic data. The 24 songs were used as the unit of analysis.

### Surveys

After each song, participants were asked to rank how much they liked the song (1 to 10), if they would replay the song (0, 1), recommend the song to their friends (0, 1), if they had heard it previously to assess familiarity (0, 1), and if they found the song offensive (0, 1). We also showed participants lyrics from the song and lyrics created by the researchers and asked them to identify the song lyrics to measure their memory of the song (0, 1).

### Market data

The streaming service provided the researchers with market data from their platform. These included the number of song streams that varied from 4,000 (NLE Choppa “Dekario”) to over 32 million (“Dance Money”). Additional data included the number of streaming stations that carried the song and online likes.

### Statistical analysis

We used a sequence of statistical approaches, increasing in sophistication, to assess the predictive accuracy of neurophysiological variables. This was done so that the models can be directly compared. The analysis begins with tests of mean differences for self-report and neurophysiologic data comparing hits and flops using Student's *t*-tests (for readability, denoted “*t*-test”). Parametric relationships were examined using Pearson correlations while logistic regressions were estimated to establish predictive accuracy. Sensitivity analysis was conducted by analyzing the 1st min of data and re-assessing the likelihood of a song being a hit.

In order to improve predictive accuracy, we trained a bagged machine learning (ML) model. Bagged models are a type of ensemble ML model that tests several machine learning algorithms in an attempt to improve accuracy above that of a single model (Dietterich, 2000). Bagged models do this by taking the output of each model individually and making a prediction based on the weighted average prediction of each model. The SuperLearner package in R was used to train and test the weighted bagged models. We included common machine learning classification algorithms in the analysis, including logistic regression, k-nearest neighbors, neural nets, and support vector machines.

Logistic regression can be considered a machine learning method since it is designed as a statistical binary classifier. Support vector machines (SVMs) trains on data by fitting a hyperplane to separate classifications. These hyperplanes can be non-linear making them well-suited for neurophysiologic data. K-nearest neighbors (KNN) uses training data to create boundaries between different classification labels. It does this by iterating through each data point and using the k-nearest observations to determine boundaries for classification. Artificial neural networks (ANN) attempt to make predictions in a way that mimics the neural patterns of the brain. It takes each variable as an input and uses a series of linear and non-linear transformations to map them into outputs. These transformations are weighted using a backpropagation algorithm seeking to improve predictive accuracy (James et al., 2013).

Bagged ML maximizes predictive accuracy by comparing the predicted value of each algorithm using a training set to the actual value. It then combines algorithms by minimizing cross-validated risk (Van der Laan et al., 2007; Polley and van der Laan, 2010) weighting each one by its contribution to accuracy. The final predicted value is calculated as the sum of the predicted value for each algorithm multiplied by the derived weights,


$${Ŷ}_{i}=\overset{N}{\underset{j=\;1}{\sum }}\beta_{j}{Ŷ}_{ij},$$

where β_*j*_ is the weight of algorithm *j* and Ŷ_*ij*_ is the predicted value for song *i* by algorithm *j*. For details, see Polley and van der Laan (2010).

To find optimal parameter settings we used 5 fold cross-validation. The logistic regression used the optimal cost settings (1, 10, 100), the number of neighbors for KNN was (3, 5, 8, 10), cost (1, 10, 100) and kernel (radial, polynomial, hyperbolic tangent) were used for SVM, and an activation function (linear, softmax), while layer size (1, 5, 10), and decay (0, 1, 10) were used for the ANN. Optimal settings were identified as logit C = 1, KNN k = 3, SVM C = 10, kernel = tanh, and ANN function = softmax, layer size = 5, and decay = 1.

Small data sets are not appropriate for machine learning as they lead to high bias in their results (Vabalas et al., 2019). To address this, we created a synthetic set data with 10,000 observations using the synthpop package in R (Nowok et al., 2016). This standard automated procedure creates observations by repeatedly randomly sampling the joint distribution of the data. This technique is used when obtaining large datasets is infeasible, including analyses of computer vision (Mayer et al., 2018), sensitive information like hospital records (Tucker et al., 2020), and with unbalanced data (He et al., 2008; Luo et al., 2018). One-half of the synthetic data was used to train the bagged ML model and tune the hyperparameters. The other half of the synthetic data was used to test it. The Appendix compares the observed data to the synthetic dataset and discusses the methodology used to generate these data. Means, standard deviations and correlations were statistically identical. All participant data were used to train the models and to generate predictions.

## Results

In the following subsections, we compare the predictive accuracy of self-reported song preferences to models using neural variables. Several measures of market impact are related to self-reports and neural variables in order to provide a baseline. Significant relationships are then used to build classifiers. Neurophysiologic data are first used to estimate perhaps the simplest classifier, a logistic regression to establish a second baseline for ML model comparison. ML predictive accuracy is assessed using neural data from participants listening to complete songs as well as to data from the 1st min of each song. The latter is included to demonstrate the robustness of our results. Analyses of possible overfitting using K-fold cross-validation are also reported to establish model appropriateness. In is important to note that only two neurophysiologic measures served as the foundation for the ML models. This streamlines the interpretation of the findings and reduces the likelihood of overfitting.

### Self-report

Self-reported liking was statistically related to the number of streams (*r* = 0.54, *N* = 24, *p* = 0.002) when analyzing participants who were familiar with the songs. Liking was not predictive for stations (*r* = −0.08, *N* = 24, *p* = 0.701) or online likes (*r* = 0.38, *N* = 24, *p* = 0.060). When analyzing songs that were unfamiliar to participants, the relationship between likes and online streams disappeared (β = −0.13, *N* = 24, *p* = 0.387). This suggests an endogeneity problem: are liked songs familiar or are more familiar songs liked? In order to avoid this issue, we analyzed data only from songs with which participants were unfamiliar. These data were aggregated to the song level and were used for all subsequent analyses.

For unfamiliar songs, self-reported liking was statistically identical for hits and flops (*M*_*hit*_ = 4.49, *M*_*flop*_ = 4.48; *t* (22) = −00.05, *p* = 0.963, *d* = −00.02). The same held for recommend [*t* (22) = −00.21, *p* = 0.829, *d* = −00.09], offensive [*t* (22) = −00.44, *p* = 0.664, *d* = −00.18] and lyrics [*t* (20) = −00.58, *p* = 0.571, *d* = −00.25]. None of the self-report measures correlated with steams (*r* = 0.16, *p* = 0.46), stations (−00.31, *p* = 0.140), or online likes (*r* = 0.05, *p* = 0.980).

### Neurophysiologic responses

Hit songs had higher immersion than flops (*M*_*hit*_ = 4.17, *M*_*flop*_ = 4.10; *t* (22) = −02.34, *p* = 0.028, *d* = −00.95) while neurologic retreat and peak immersion did not differ between hits and flops (retreat: *t* (22) = 2.01, *p* = 0.057, *d* = 0.82; peak: *t* (22) = −00.14 *p* = 0.887, *d* = −00.06). Immersion was not correlated with streams (*r* = 0.03, p = 0.870), nor was peak immersion (r = 0.26, p = 0.202), while neurologic retreat trended toward significance (*r* = −00.39, *p* = 0.057). Immersion was negatively related to age (*r* = −00.31, *p* < 0.001) and varied by gender (Male: 4.06, Female: 4.20; *t* (650) = −03.04 *p* < 0.001, *d* = −00.26).

### Accuracy

Logistic regression models were used to assess whether neurophysiologic measures could predict hits. Model 1 only included immersion. Then Model 2 added retreat to test if it would improve accuracy. Both Model 1 and Model 2 were significantly better at predicting hits than chance (*F* = 5.48, *p* = 0.023; *F* = 3.27, *p* = 0.05). Model 1 and Model 2 correctly classified hits and flops 66% and 70% of the time, respectively. Model 2 classified hits with 69% accuracy and flops with 62% accuracy using only the two neurophysiologic variables (ps < 0.001). While retreat and immersion are correlated with each other (*r* = −0.51, *p* = 0.011), Model 2 did not suffer from multicollinearity (VIF = 1.07). The results were robust to the inclusion of an indicator for offensive lyrics (*p* = 0.68). As expected the neurophysiologic variables were not statistically related to the self-reported desire to replay the song, recommend the song to others, or the number of online likes for each song (ps > 0.68).

### Machine learning

A bagged ML model was trained on one-half of the synthetic data using immersion and retreat as independent variables. The ML approaches that contribute more have a higher coefficient and lower risk (Polley and van der Laan, 2010). The k-nearest neighbor model contributed the most (coef = 0.98, risk = 0.018) followed by a neural net (coef = 0.012, risk = 0.18). A logistic regression and support vector machines contributed nothing to an accurate classification.

**Figure 2:**
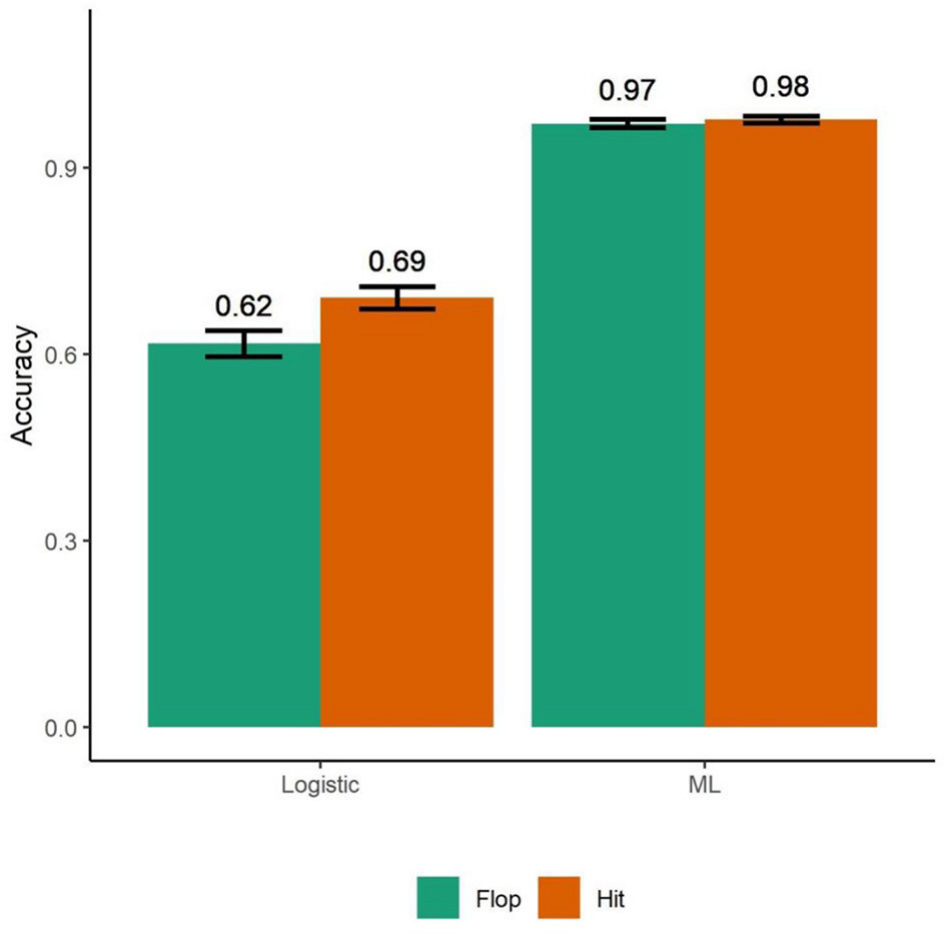
A traditional logistic regression model using neurophysiologic immersion and retreat as independent variables correctly classified hits with 69% accuracy and flops with 62% accuracy (*N* = 5,000). A bagged machine learning model using the same two independent variables had accuracy of 96.6% in classifying hits while flops were classified with 97.6% accuracy. Bars are standard deviations.

The bagged ML model was able to accurately classify the type of song 97.2% of the time. This statistically greater than the base rate (Successes = 4,800, *N* = 5,000, *p* < 0.001) using the exact binomial test (Hollander and Wolfe, 1973). Examining specificity and sensitivity, hits were classified correctly 96.6% of the time and flops were classified with 97.6% accuracy (Figure 2).

Next, we assessed the bagged ML model's ability to predict hits from the original 24 song data set. The bagged ML model accurately classified songs with 95.8% which is significantly better than the baseline 54% frequency (Success = 23, *N* = 24, *p* < 0.001). Only one song, Evil Spider, was classified incorrectly. This song was a flop with nearly 54,000 streams but was classified as a hit due to its high immersion.

We conducted a bootstrap procedure with 1,000 iterations on both the bagged ML model and the logistic model to compare their accuracy for hits and flops. The logit was trained on one data set (*N* = 5,000) and then assessed for accuracy on another set of data (*N* = 5,000) for each iteration. The bagged ML model predicted hits ML: CI = [1, 1]; Logistic: CI = [0.67, 0.73]; *t* (1,998) = −115.86, *p* < .001) and flops (ML: CI = [0.82,1.00]; Logistic: CI = [0.59, 0.63]; *t* (1,998) = −121.13, *p* < 0.001) better than the logistic model.

**Figure 3:**
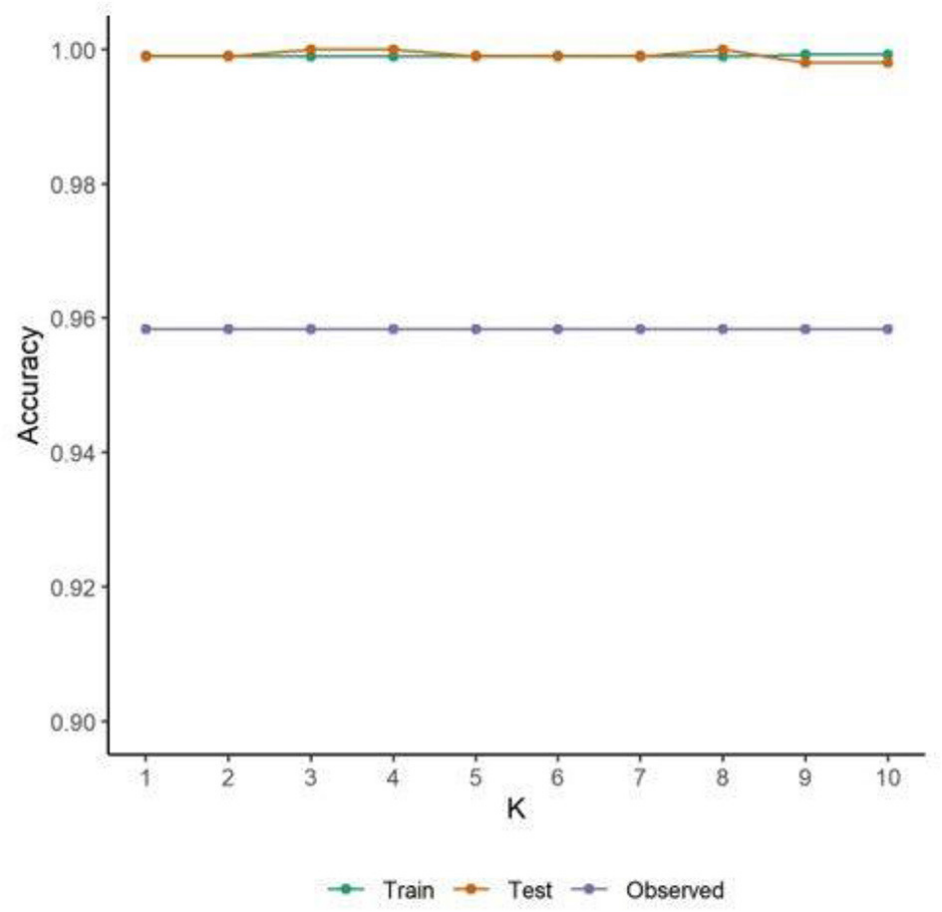
A 10-fold cross validation indicates the bagged ML model consistently predicts test data with ~99% accuracy. Accuracy for the observed data is consistent at ~96% indicating that the model does not overfit the data.

The model was assessed for overfitting by running a 10-fold cross validation on the bagged ML and comparing the predictive accuracy of the training set, test set, and observed data. This analysis shows that the bagged ML does not appear to overfit the test data as the accuracy is high and consistent (James et al., 2013). As expected, the accuracy is higher on the training and test synthetic data across the k-folds (~0.99) compared to the *N* = 24 observed data (~0.96). Nevertheless, across the three data sets the accuracy of the model is high, similar, and consistent (Figure 3).

### 1 min of data

To establish the robustness and practical applications of our findings, we analyzed the accuracy of neurophysiology collected from the first 1 min of data to identify hits. We ran the same logistic and bagged ML models described previously using only the data from the 1st min of each song. We did not find a significant relationship between immersion (OR = 362.25, *N* = 24, *p* = 0.101) or retreat (OR = 27175.63, *N* = 24, *p* = 0.406) and hit songs. However, using immersion and retreat, we were able to correctly classify songs 66% of the time using a logistic regression. Specificity and sensitivity were moderate at 77% and 56%, respectively.

**Figure 4:**
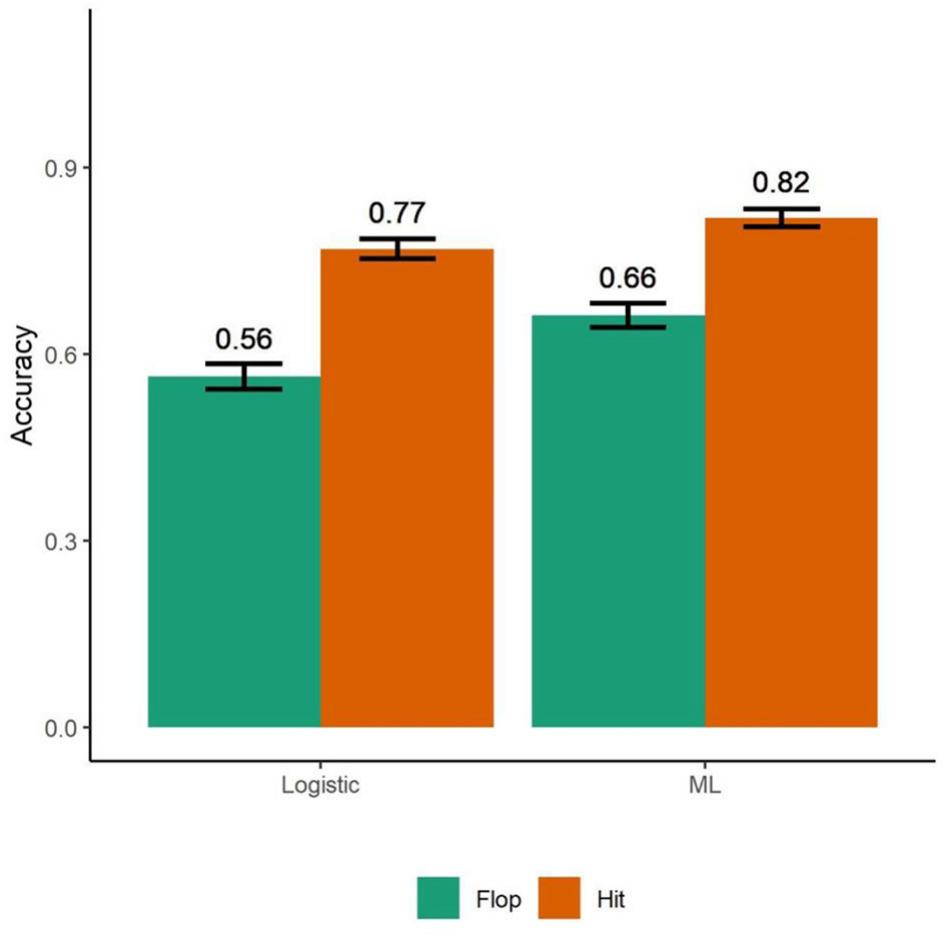
A logistic regression model trained on neurophysiologic responses for the 1st min of songs correctly classified hits with 77% accuracy and flops with 56% accuracy (*N* = 10,000). The bagged machine learning model classified 74% of songs correctly. Hits were classified with 82% accuracy while flops were identified accurately 66% of the time. Model training used half of the synthetic data set (*N* = 5,000) using a bootstrapped evaluation of 5,000 observations per iteration for 1,000 iterations. Bars are standard deviations.

We also created another synthetic data set to train a bagged machine learning model. Our bagged ML model had overall accuracy of 74%. It predicted hit songs with 82% accuracy and flops with 66% accuracy (Figure 4). Using the bagged ML model on the original data, we found that it was able to predict hits and flops 66% of the time. Bootstrapping the results, the bagged ML model outperformed the logistic model in classifying hit songs (ML: CI = [0.80, 0.82], Logistic: CI = [0.75, 0.7848]; *N* = 5,000, *t* (1,998) = −41.76, *p* < 0.001), and flops (ML: CI = [0.65, 0.70], Logistic: CI = [0.54, 0.58]; *t* (1,998) = −22.61, *p* < 0.001).

## Discussion

The key contribution of the present study is to demonstrate that neurophysiologic measures accurately identify hit songs while self-reported “liking” is unpredictive. In addition, we showed that neurophysiology, combined with machine learning, substantially improves the classification of hit songs when compared to linear statistical models. Our goal was to provide a methodology that other researchers can use to predict hit songs of different genres, in different geographic locations, and for study populations with different demographics. The approach described here should also be tested for its ability to accurately predict hits for other forms of entertainment that are known to be difficult to ascertain, including movies, TV shows, and social media posts in order to confirm and extend our results. Indeed, our use of a commercial neuroscience platform makes such extensions feasible for non-neuroscientists.

Forecasting one's own behavior based on reflection is fraught and using self-report to predict market outcomes of entertainment is nearly always a fool's errand (Sheeran, 2002; Woodside and Wilson, 2002; Brenner and DeLamater, 2016). Even experts cannot identify high quality goods and services from their imitators (Ashenfelter and Jones, 2013; Almenberg et al., 2014). While people want to hear new music, they prefer music that is similar to familiar songs generating a bias in self-reports (Ward et al., 2014). The Hit Song Science problem is typically addressed by mining very large datasets (McFee et al., 2012). We took a different approach, collecting neurophysiologic data from a moderate number of people to predict aggregate outcomes. We showed that self-reported liking only identifies hit songs if one was already familiar with the song. This is most likely due to endogeneity: participants are more likely to report they like a song if they have heard it often. Once we removed participants' familiar songs, self-reported liking ceased to predict hits.

The use of neurophysiologic data to predict aggregate outcomes is an approach that has been labeled “brain as predictor” or “neuroforecasting.” This approach captures neural activity from a small group of participants to predict population outcomes (Berkman and Falk, 2013; Dmochowski et al., 2014; Genevsky et al., 2017). Neurologic data have been shown to predict outcomes more accurately than self-reports for sunscreen use, smoking reduction strategies, watching TV, and crowdfunding requests (Falk et al., 2010, 2011; Yang et al., 2015; Genevsky et al., 2017; Zak, 2022). While estimating predictive models using neural data is an improvement over poorly-predicting self-reported measures, the accuracy of neural forecasts have generally been no better than 50%. The closest published study to the report here used fMRI data from 28 people to predict the popularity of 20 songs. One brain region, the nucleus accumbens, was correlated with aggregate outcomes but was only able to correctly classify hits with 30% accuracy (Berns and Moore, 2012).

Our analysis showed that two measures of neurophysiologic immersion in music identified hits and flops with 69% accuracy using a traditional linear logistic regression model. A logistic regression using only the 1st min of the song was nearly as accurate at 66% and was 77% accurate at classifying which songs were hits. This is a substantial improvement over the existing literature. Most of the models cited above using neural data to predict aggregate outcomes have focused on attentional responses. The neurophysiologic data we used convolves attentional and emotional responses and this may account for our improved predictive accuracy (Lench et al., 2011; Zak and Barraza, 2018; Zak, 2020). Emotional responses are a key component of persuasive communication because emotions capture the subjective value of an experience (Barraza et al., 2015; Cacioppo et al., 2018; Falk and Scholz, 2018; Doré et al., 2019). The analysis here indicates that emotional responses also appear to determine which songs become hits.

Applying a bagged machine learning model to neural data improved its predictive accuracy from 69% to 97%. We also demonstrated the robustness and practical use of our approach by correctly classifying hits with 82% accuracy using the 1st min of songs. It is worth noting that no demographic or self-report data were used in these models. Further, our findings are unlikely due to chance. Machine learning using neural data has been used to identify mental illness (Stahl et al., 2012; Khodayari-Rostamabad et al., 2013; Amorim et al., 2019), epilepsy (Shoeb and Guttag, 2010; Buettner et al., 2019), stress (Subhani et al., 2017), and to recognize emotions (Zhang et al., 2020). Market researchers have applied machine learning to neural data to predict views of Superbowl ads and behavioral responses to advertising (Guixeres et al., 2017; Wei et al., 2018). As of this writing, machine learning models of music have used lyrical content rather than neural data to classify hits with only moderate accuracy (Dhanaraj and Logan, 2005; Singhi and Brown, 2014). We extended these approaches by using neural responses to music from a modest number of people to identify hits. Future work could connect neural responses to lyric classifications for additional insights.

Rather than choose a single machine learning algorithm, our use of an ensemble model eliminated a manual search for the best approach. The analysis showed that a k-nearest neighbors' (KNN) algorithm was responsible for the majority of the explanatory power. While machine learning models have been called “black boxes,” our analysis showed that hit songs have higher immersion than flops and do so with a large effect size (*Cohen's d* = *0.95*). Hits also produced less neurologic retreat than flops with a similar effect size (*Cohen's d* = *0.82*). Another reason to use machine learning to classify hits is that neurophysiologic data are inherently non-linear. Unlike logistic regressions, KNN's incorporate non-linear relationships making it ideally suited to neural data.

It is noteworthy that only three neurophysiologic variables were used in the analysis. Among these, only two of the variables, immersion and retreat, were statistically associated with classifying hits. The results are consistent with the intuition that hits are expected to have higher immersion and produce less retreat than do flops. The parsimony of models with two variables, and their associated high accuracy, supports the measurement of peripheral neural measures for this application. While the classification accuracy using linear relationships of these variables, as in the logistic regression we reported, was only moderate, the ML approach utilized non-linear relationships which are more appropriate for neural responses (Timme and Lapish, 2018).

While the accuracy of the present study was quite high, there are several limitations that should be addressed in future research. First, our sample was relatively small so we are unable to assess if our findings generalize to larger song databases. The large effect sizes indicate the results are likely to be similarly accurate if other songs were tested. We also created a synthetic data set to train the machine learning model. These data, while generated from human neural responses, may have overweighted subtle relationships not evident in the original data. Nevertheless, this approach has become standard when access to large samples, such as in experimental studies as reported here, is not available (Hoffmann et al., 2019). The use of synthetic data allows researchers to gather less direct participant data with a small or no loss in accuracy. While we found high accuracy using the observed data, we did not have access to an outside sample of songs to validate the model further. This means our model might have overfitted the data.

## Conclusion

Measuring emotional responses using neuroscience technologies provides a new way for artists, record producers, and streaming services to delight listeners with new music. Our contribution is to show that omnibus neuroscience measurements from the peripheral nervous system quite accurately classify hits and flops. Rather than asking users if they “like” a new song, wearable neural technologies, like those in this study, could assess the neural value of content automatically. Steaming services' “Discover Weekly,” and “Personalized Soundtracks” could more effectively build playlists of desired new music by measuring neurologic immersion when users listen to just the 1st min of a new song. Music from users' existing playlists could also be chosen by using neurologic immersion to identify mood states as was recently shown (Merritt et al., 2022). Our findings, if replicated, indicate that as neuroscience technologies enter into general use, the ability to curate music and other forms of entertainment to give people just what they want will improve existing recommendation engines benefiting artists, distributors, and consumers.

## Data availability statement

The datasets presented in this study can be found in online repositories. The names of the repository/repositories and accession number(s) can be found below: https://doi.org/10.3886/E152561V1.

## Ethics statement

The studies involving human participants were reviewed and approved by IRB Claremont Graduate University. The patients/participants provided their written informed consent to participate in this study.

## Author contributions

PZ designed the study. SM and KG performed the statistical analysis. SM, KG, and PZ wrote the manuscript. All authors discussed the results and provided critical feedback and helped shape the research, analysis and manuscript.
