# Supplementary material for: Accurately predicting hit songs using neurophysiology and machine learning
Source: Front Artif Intell. 2023 Jun 20;6:1154663. doi: 10.3389/frai.2023.1154663 (PMC10318137; doi:10.3389/frai.2023.1154663)
Supplement: Supplementary file 1 [file Data_Sheet_1.docx]

**Appendix**

The parameters used for synthetic data generation were standard, including minimum levels (2), seed (1), and smoothing (spline).  The number of observations was set to 10,000. The statistical tests in the table below shows that the synthetic data closely match the observed data on means, variances, and correlations. For transparency, plots are also shown revealing some variation between synthetic and observed data. However, given the small sample of the observed data this is expected.

*Figures A1-A3 show the distribution of the data with the observed data in black and the synthetic data in red.*


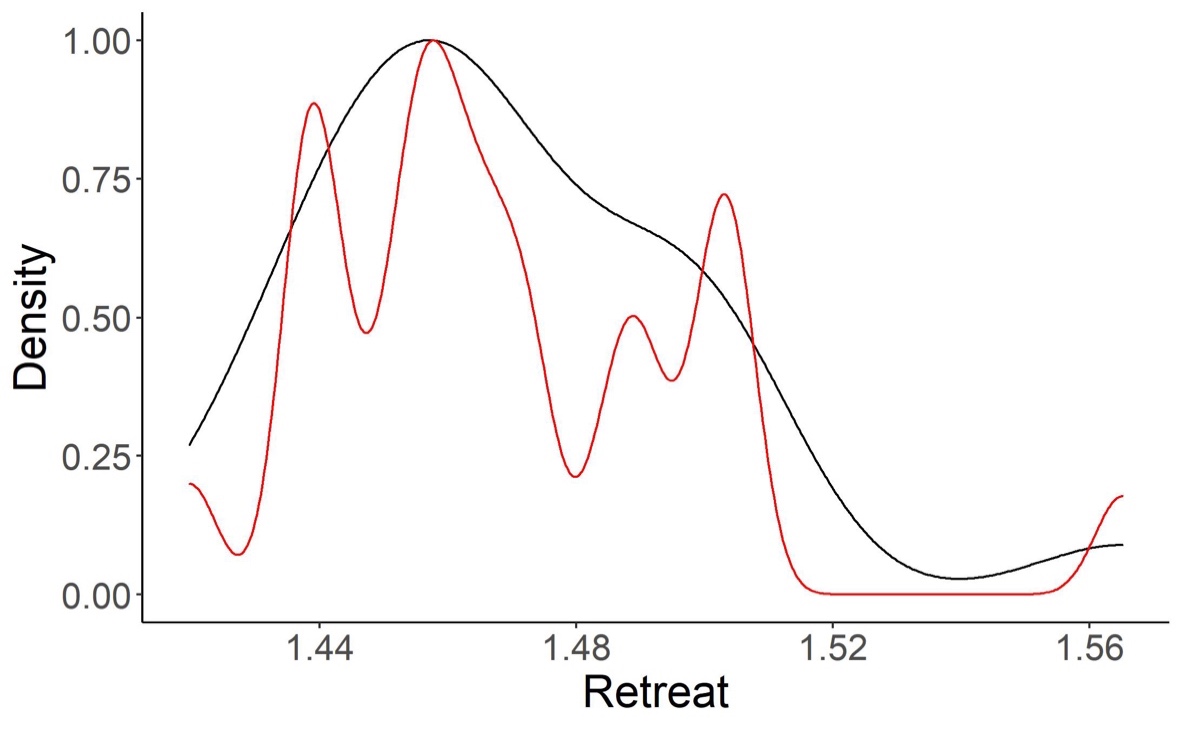

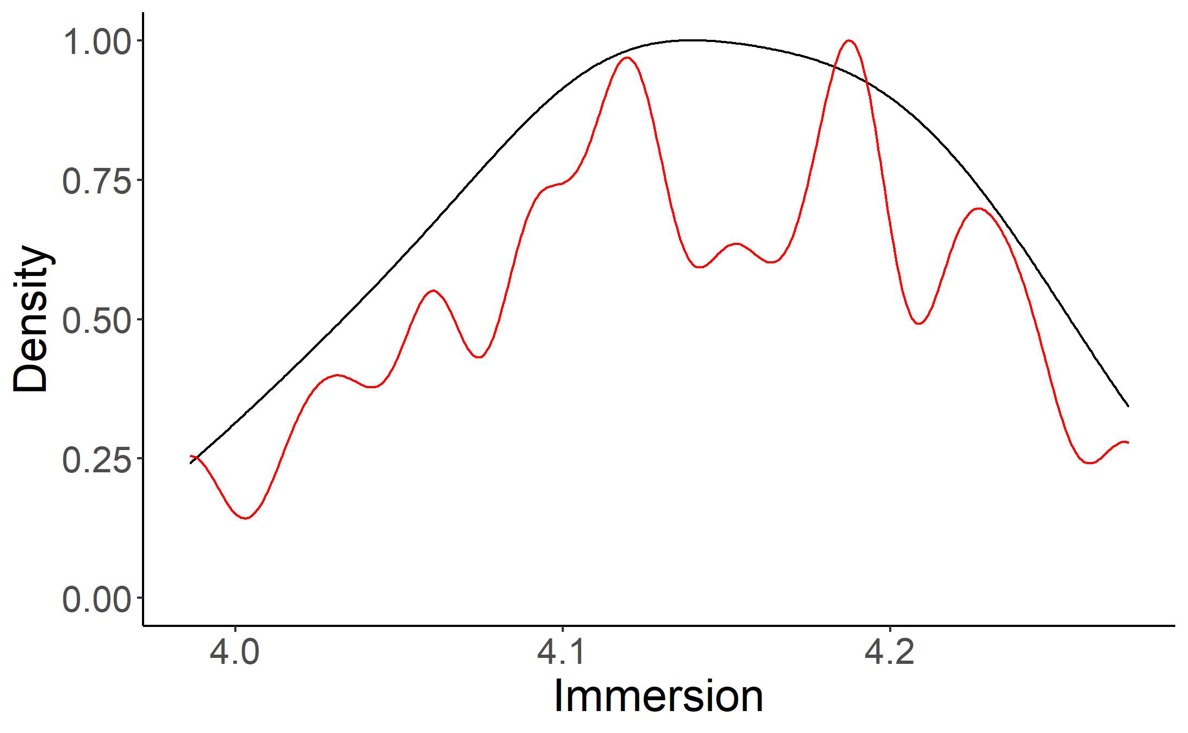

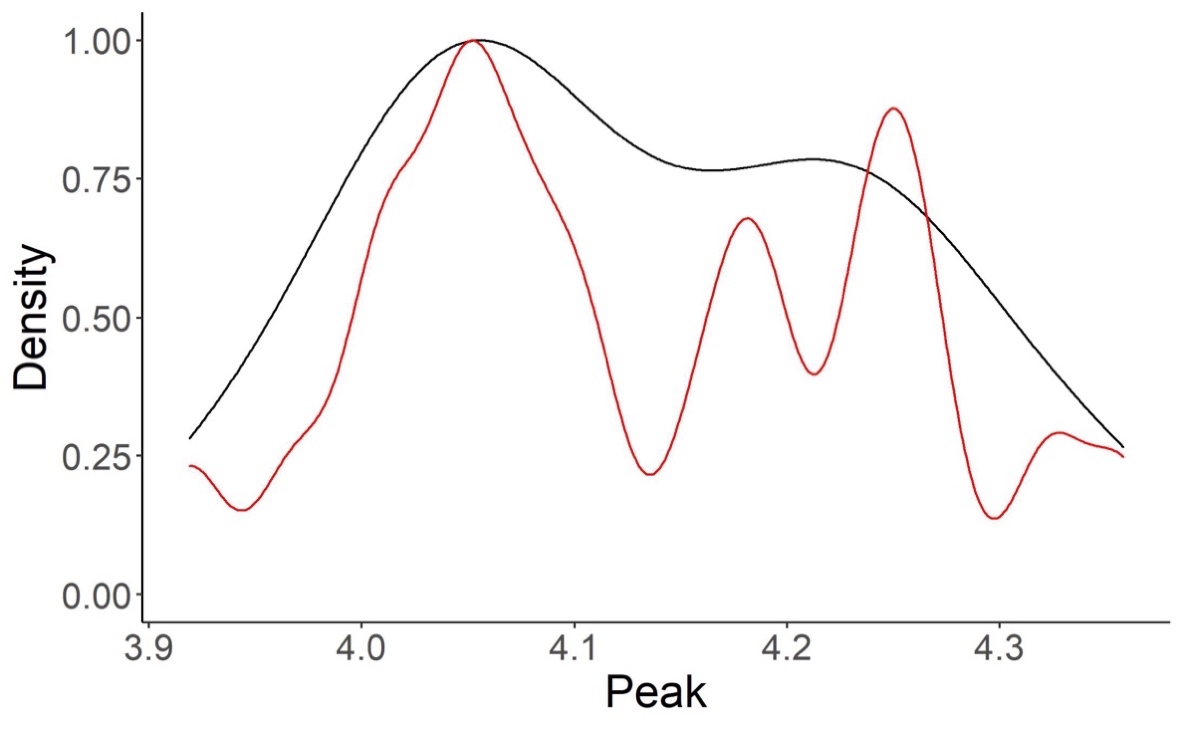


*Table A1: Comparison of summary statistics for the observed and synthetic datasets.*

|  |  | N | Mean | S.D. | r_hit | r_immersion | r_peak | r_retreat |
| --- | --- | --- | --- | --- | --- | --- | --- | --- |
| Hit | Observed | 24 | 0.54 | 0.51 | - |  |  |  |
|  | Synthetic | 10000 | 0.54 | 0.49 | - |  |  |  |
| Immersion |  |  |  |  |  |  |  |  |
|  | Observed | 24 | 4.13 | 0.08 | 0.44 | - |  |  |
|  | Synthetic | 10000 | 4.14 | 0.07 | 0.44 | - |  |  |
| Peak |  |  |  |  |  |  |  |  |
|  | Observed | 24 | 4.13 | 0.12 | 0.03 | -0.11 | - |  |
|  | Synthetic | 10000 | 4.13 | 0.12 | -0.02 | -0.01 | - |  |
| Retreat |  |  |  |  |  |  |  |  |
|  | Observed | 24 | 1.47 | 0.03 | -0.39 | -0.51 | -0.18 | - |
|  | Synthetic | 10000 | 1.47 | 0.03 | -0.11 | -0.37 | -0.23 | - |

No significant differences were found between the observed and synthetic data for means (t-tests), standard deviations (F-tests), or correlations (t-tests) as shown in Table A1. P-values for each test are shown in Table A2.

|  |  | Mean | SD | r_hit | r_immersion | r_peak | r_retreat |
| --- | --- | --- | --- | --- | --- | --- | --- |
| Hit |  |  |  |  |  |  |  |
|  | Statistic | -.04 | 1.05 | - |  |  |  |
|  | df | 23.11 | 23,9999 | - |  |  |  |
|  | p | .969 | .804 | - |  |  |  |
| Immersion |  |  |  |  |  |  |  |
|  | Statistic | -0.04 | 1.04 | 0.00 | - |  |  |
|  | df | 23.11 | 23,9999 | 10022 | - |  |  |
|  | p | .971 | .816 | 1.00 | - |  |  |
| Peak |  |  |  |  |  |  |  |
|  | Statistic | -0.04 | 1.04 | 1.06 | -0.46 | - |  |
|  | df | 23.11 | 23,9999 | 10022 | 10022 | - |  |
|  | p | .970 | .816 | .291 | .649 | - |  |
| Retreat |  |  |  |  |  |  |  |
|  | Statistic | 0.13 | 1.08 | -1.37 | -0.79 | 0.24 | - |
|  | df | 23.10 | 23,9999 | 10022 | 10022 | 10022 | - |
|  | p | .896 | .707 | .172 | .429 | .813 | - |

We established two baselines for comparison to the bagged ML model. The first is the base rate probability of selecting a hit song. In both the synthetic and observed data this is 54%. The second baseline was established by taking the dominant observed outcome in the data (hits) and predicting how many hits in the test set. This baseline was 68%. The figures below compare the hit and miss rates for all three neurophysiologic variables for the original data and synthetic data.

*Figures A4-A6: A comparison of the hit and miss rate for each neurophysiologic variable for the observed and synthetic datasets. Means, standard deviations, and ranges (25th and 75th percentiles*) *are shown.*

**
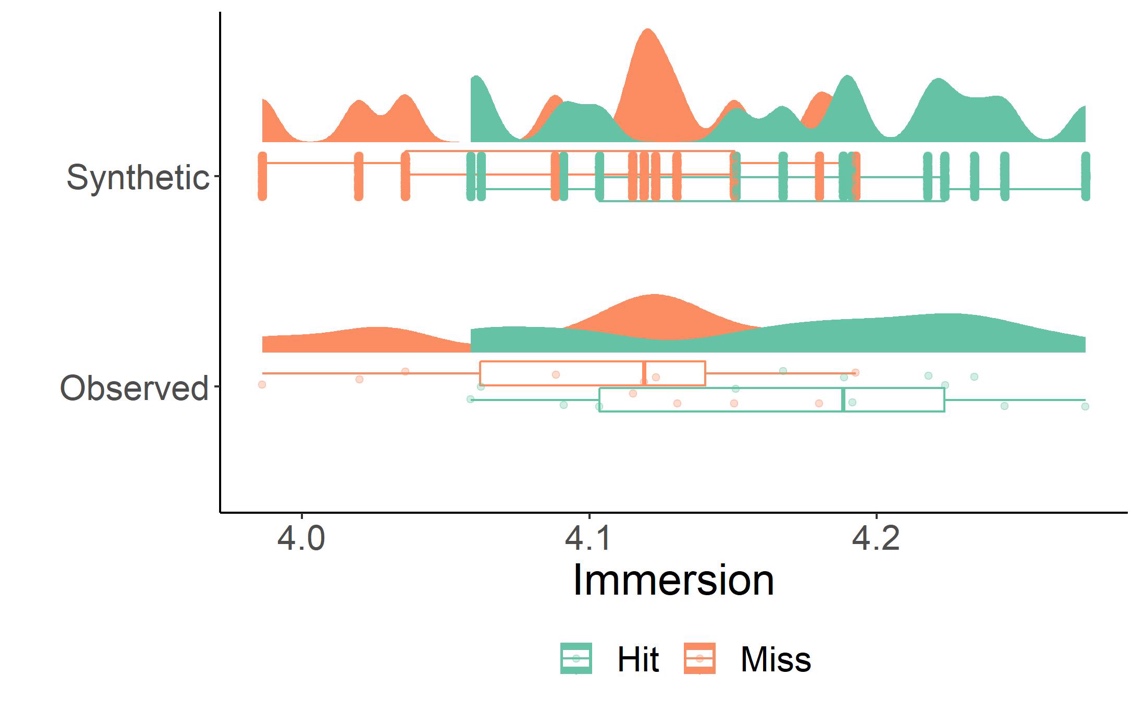

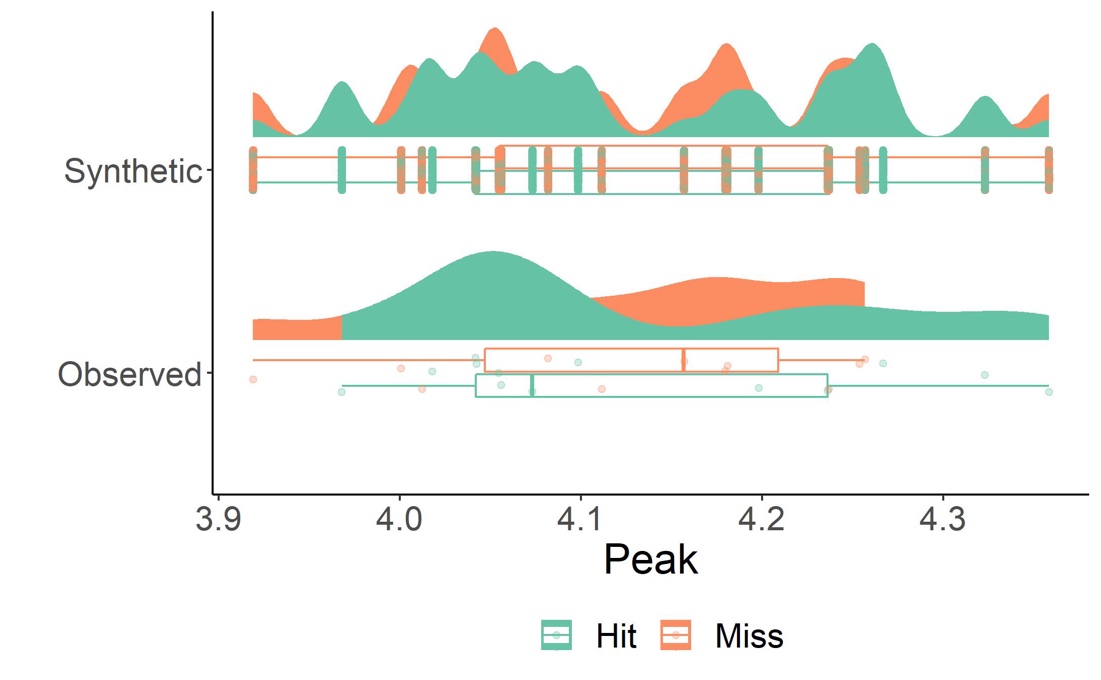

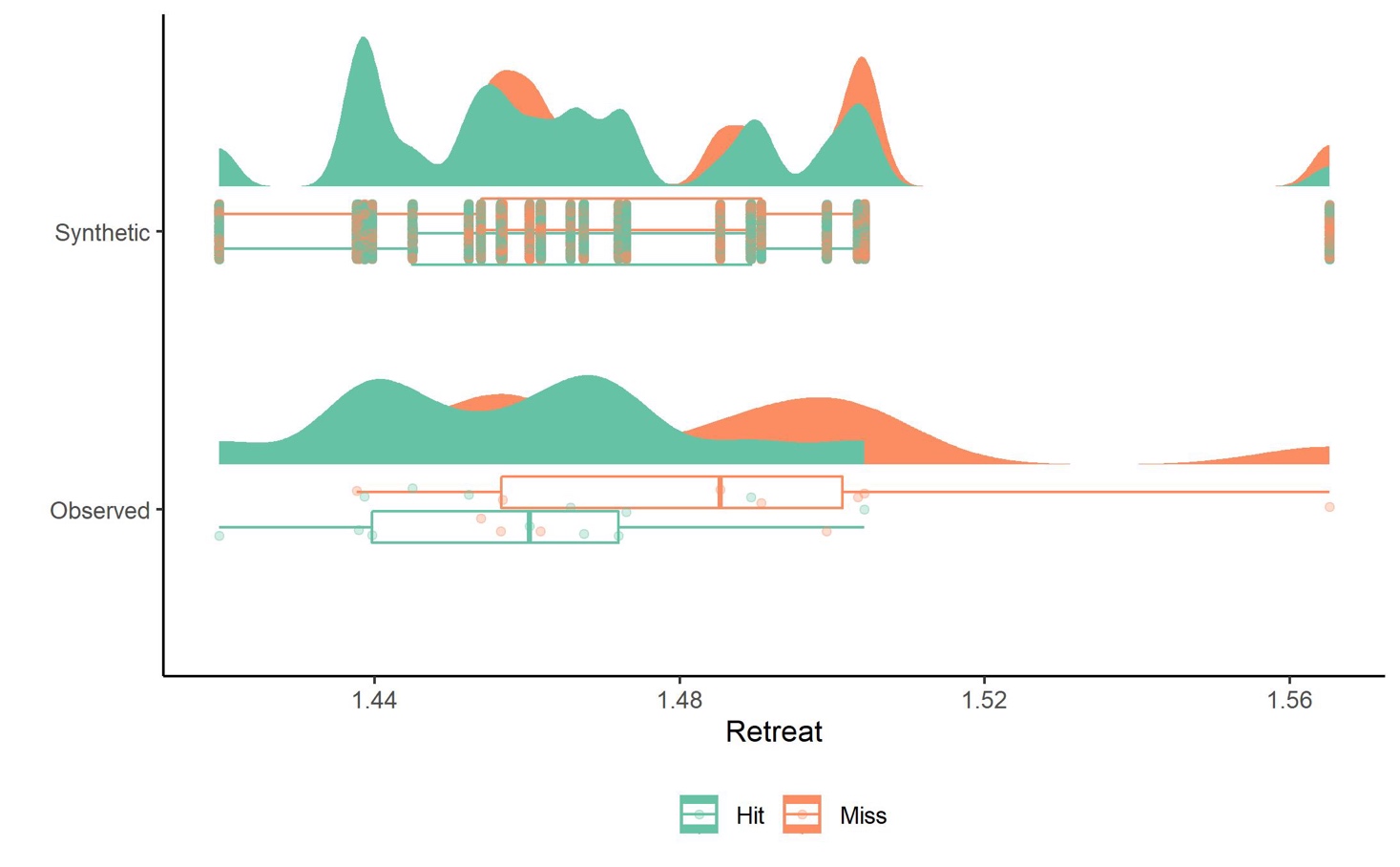
**
